# Combined pulsed field ablation and left atrial appendage occlusion in a patient with persistent atrial fibrillation and adenomyosis-related menorrhagia: case report and 1-year follow-up

**DOI:** 10.1093/ehjcr/ytag353

**Published:** 2026-05-20

**Authors:** Sophia Alexiou, Athanasios Samaras, Georgios Stavropoulos, Apostolos Tzikas, Nikolaos Fragakis

**Affiliations:** 2nd Department of Cardiology, Hippokration General Hospital, Aristotle University of Thessaloniki, 49 Konstantinoupoleos Street, Thessaloniki 54642, Greece; 2nd Department of Cardiology, Hippokration General Hospital, Aristotle University of Thessaloniki, 49 Konstantinoupoleos Street, Thessaloniki 54642, Greece; 2nd Department of Cardiology, Hippokration General Hospital, Aristotle University of Thessaloniki, 49 Konstantinoupoleos Street, Thessaloniki 54642, Greece; 2nd Department of Cardiology, Hippokration General Hospital, Aristotle University of Thessaloniki, 49 Konstantinoupoleos Street, Thessaloniki 54642, Greece; 2nd Department of Cardiology, Hippokration General Hospital, Aristotle University of Thessaloniki, 49 Konstantinoupoleos Street, Thessaloniki 54642, Greece

**Keywords:** Atrial fibrillation, Pulsed field ablation, Left atrial appendage closure, Combined procedure, Case report

## Abstract

**Background:**

Pulsed field ablation (PFA) is an emerging modality for atrial fibrillation management, offering rapid pulmonary vein isolation and the possibility of the left atrial posterior wall ablation. Combining PFA with left atrial appendage occlusion (LAAO) represents a holistic strategy for managing heart rhythm abnormalities and minimizing stroke risk, particularly in patients with high thromboembolic and bleeding risks.

**Case summary:**

We present a case of a 52-year-old woman with persistent atrial fibrillation, recent percutaneous coronary intervention, and a history of adenomyosis-related menorrhagia, exacerbated by the use of dual antithrombotic therapy. She underwent a single-session PFA and LAAO. The procedure was uncomplicated. At three-month follow-up, imaging confirmed appropriate device placement and complete closure. Anticoagulation was discontinued at three months, and the patient remained asymptomatic at 1-year follow-up while on single antiplatelet therapy.

**Discussion:**

This case highlights the feasibility and safety of combining PFA and LAAO in a high-risk patient and underscores the potential for broader adoption in selected populations. Larger studies are needed to evaluate long-term outcomes.

Learning points

- Combining pulsed field ablation (PFA) with left atrial appendage occlusion (LAAO) in a single session is safe and effective for AF patients with bleeding contraindications to anticoagulation.
- Adenomyosis-related menorrhagia, refractory or contraindicated for hormonal therapy, requires multidisciplinary care prioritizing LAAO for stroke prevention.
- PFA-first sequence optimizes single transseptal access and avoids catheter-device interaction in anatomically challenging LA-PV-LAA configurations.

## Introduction

Pulsed field ablation (PFA) is an established non-thermal modality for atrial fibrillation (AF), enabling rapid pulmonary vein isolation (PVI) with minimal collateral tissue injury and including occasionally lesions on the left atrial posterior wall (LAPW).^1–3^ For patients with elevated thromboembolic risk who cannot tolerate long-term anticoagulation, left atrial appendage occlusion (LAAO) provides a safe and effective alternative to stroke prevention.^4^ The OPTION trial recently demonstrated that combining Watchman LAAO with AF ablation yields stroke and systemic embolism rates comparable to anticoagulation, while significantly reducing non–procedure-related bleeding complications.^5^ This evidence suggests that combining PFA and LAAO in a single session may enhance efficiency and outcomes in selected high-risk patients. We present the case and 1-year follow-up of a patient with persistent AF and bleeding contraindications who underwent this combined strategy.

## Case presentation

A 52-year-old woman with episodes of persistent AF, arterial hypertension and percutaneous coronary intervention (PCI) three months ago due to an acute myocardial infarction (MI) was admitted for scheduled PFA of AF. She underwent radiofrequency (RF) ablation a year ago, which was initially deemed successful with complete PVI and confirmed entrance and exit block. However, AF recurred six months later. The patient also reported recurrent menorrhagia while on rivaroxaban, which was exacerbated by the addition of clopidogrel due to recent PCI. Menorrhagia was attributed to adenomyosis as determined by gynaecology consultation, while hormonal therapies were deemed contraindicated due to the recent coronary stenting and elevated thrombotic risk. This condition has led to several episodes of severe anaemia, with haemoglobin levels around 6.5 g/dL and haematocrit 21%, necessitating hospitalization and blood transfusions.

**Figure 1:**
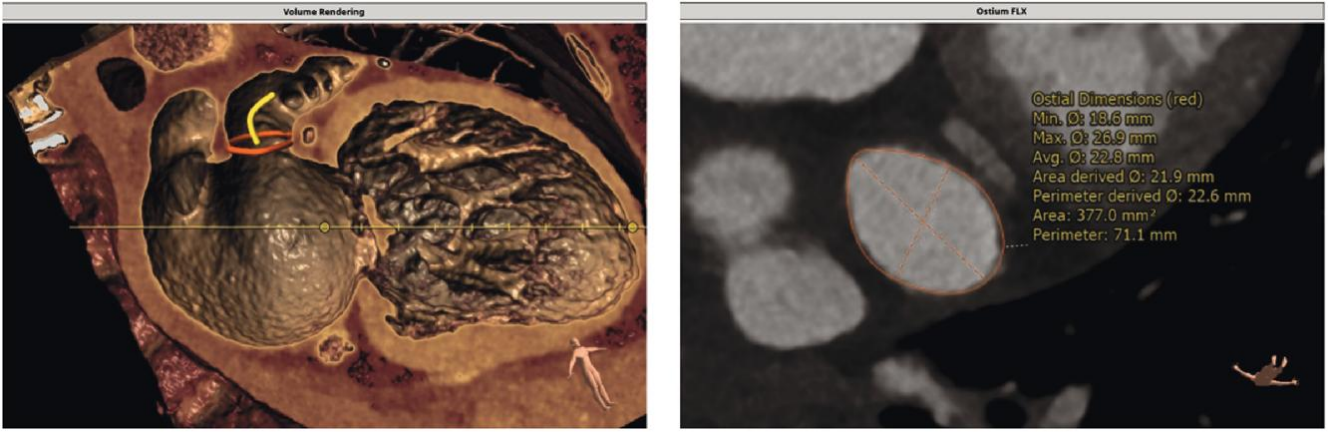
Pre-procedural planning with cardiac computed tomography to define the anatomy and size of the left atrial appendage.

The patient’s CHA_2_DS_2_-VASc score of 3, suggesting a moderated annual thromboembolic risk of 3.2% and HAS-BLED score of 3, corresponding to a comparable annual bleeding risk of 3.7% in anticoagulated patients, categorized the patient as high risk for long-term anticoagulation.^6,7^ A multidisciplinary discussion between cardiology, electrophysiology, and gynaecology specialists, determined the clinical plan to perform single-session PFA and LAAO, followed by temporary dual antithrombotic therapy (apixaban + clopidogrel) during the device endothelialization period (1–3 months), with later transition to lifelong single antiplatelet therapy (clopidogrel). PFA was preferred over focal RF for repeat PVI due to (1) shorter left atrial dwell time (LADT) (∼20–30 min), (2) compatibility with a single transseptal puncture optimized for LAAO, and (3) reduced risk of RF-related tissue injury in a patient with significant bleeding history. Pre-procedural cardiac computed tomography (CT) confirmed suitable left atrial appendage anatomy and absence of thrombus (*Figure 1*).

**Figure 2:**
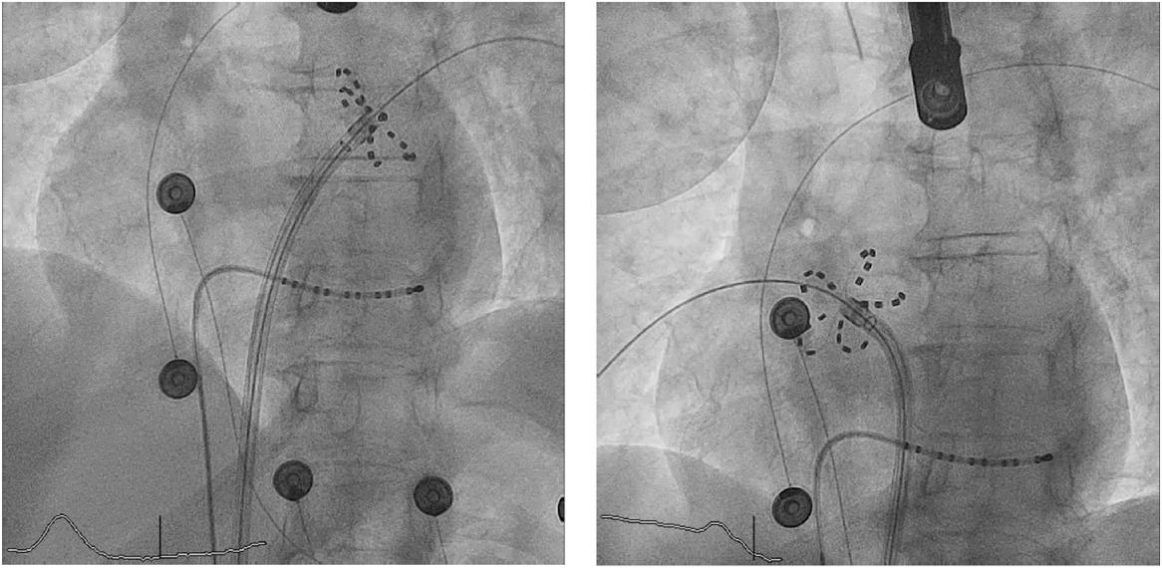
Panels (*A–B*) show, in LAO view, the position of the penta-spline ablation catheter, in flower-like configuration, during ablation of the left superior pulmonary vein (*A*) and the right inferior pulmonary vein (B).

Under general anaesthesia, a single transseptal puncture was performed using a BRK needle and SL0 sheath, under transoesophageal echocardiography (TOE) guidance, in the inferior/mid part of the interatrial septum. LAA thrombus was ruled out and LAA dimensions were evaluated on both pre-procedural CT and intraprocedural TOE prior to PFA. The SL0 was then exchanged for a 15F Faradrive steerable sheath. A penta-spline ablation catheter (Farawave, Farapulse, Boston Scientific, USA) was advanced in the left atrium for systematic pulmonary vein (PV) ostial positioning, and potentials were recorded, thus indicating that all 4 PVs were reconnected. . We chose not to prioritize the use of high-resolution 3D mapping since no organized atrial tachycardias were observed during the episodes of recurrence, aiming to minimize LADT and procedural complexity in this high-bleeding-risk patient especially since the combined PFA and LAAO procedure was anticipated to further prolong the LADT. Electrical isolation of all PVs was achieved using the standard PFA protocol (4 basket- and 4 flower-mode applications per vein), followed by 12 flower-mode lesions targeting the LAPW (*Figure 2*).

**Figure 3:**
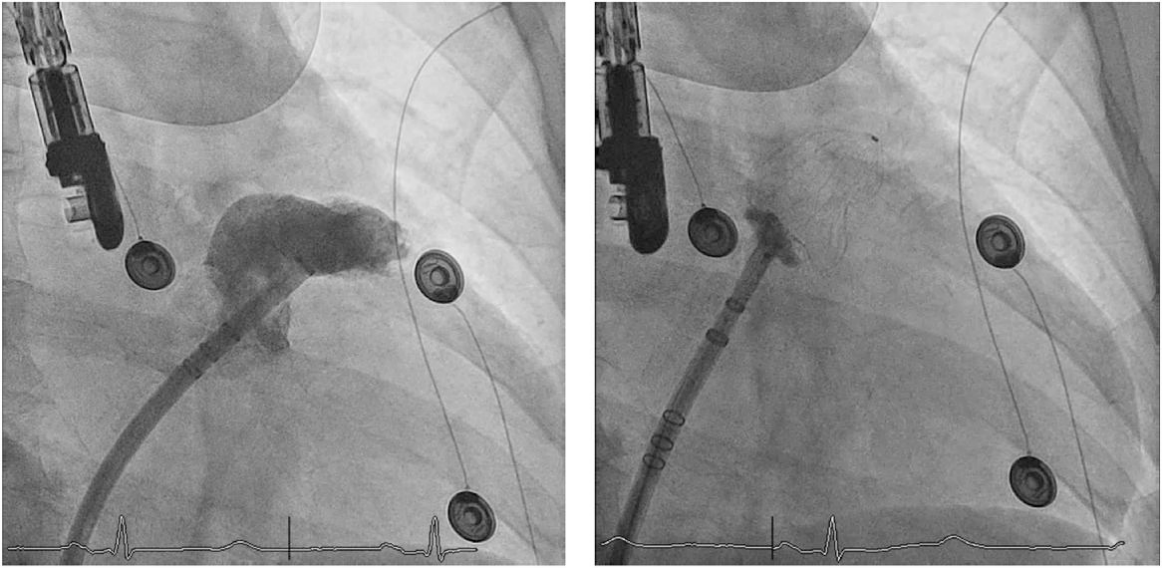
Panels (*A–B*) show in RAO view, respectively, LAA selective angiography and Watchman device placement. LAA, left atrial appendage.

Post-ablation mapping confirmed sustained PV isolation with documented exit block after pacing with the Farawave catheter. Following ablation, the sheath used for ablation was exchanged for a Watchman sheath. Two selective LAA angiograms guided by pre-procedural CT projections were performed with a 6 F pigtail catheter. A Boston Scientific Watchman FLX 31 mm device was implanted under echocardiographic and fluoroscopic guidance. Tug-test and PASS criteria confirmed device stability. Final imaging confirmed complete sealing without peri-device leak, and the device was released (*Figure 3*). Reassessment of the LAA dimensions on post-ablation TOE showed no evidence of ridge oedema or changes in LAA ostial dimensions. Femoral access sites were closed with the figure-of-8 technique.

**Figure 4:**
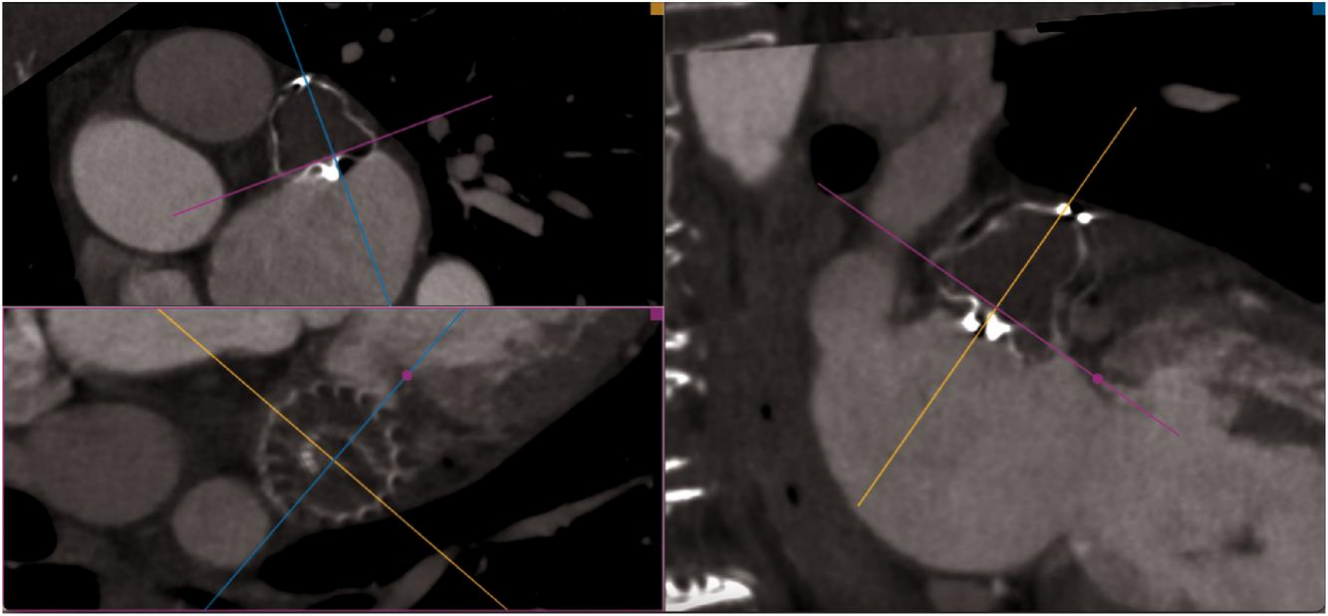
Computed tomography (CT) scan 3 months after the procedure confirmed the correct position of the device and complete LAA closure.

The patient had an uneventful recovery and was discharged the following day, after transthoracic echocardiography confirmed the absence of pericardial effusion and appropriate device positioning. At discharge, she was prescribed apixaban 5 mg twice daily in addition to continuing clopidogrel 75 mg once daily. Apixaban was selected over rivaroxaban based on institutional preference and clinical judgment favouring its observed lower bleeding risk in high-risk phenotypes, with a comparable short half-life enabling quicker transition to single antiplatelet therapy after endothelialization.^8^ This temporary dual therapy represents standard practice post-LAAO Watchman implantation (typically lasting 1 to 3 months during the device healing period). A 3-month follow-up CT scan confirmed device stability, absence of device-related thrombosis and complete LAA closure (*Figure 4*).

At 3 months, apixaban was replaced by lifelong clopidogrel. At 12-month follow-up, the patient remained asymptomatic and without documented AF episodes.

## Discussion

AF ablation combined with LAAO is gaining traction as an integrated strategy, with PFA offering distinct advantages over thermal energy in this setting, enabling rapid PVI and possible posterior wall isolation with minimal myocardial injury.^1–3^ The Farapulse™ system achieves PVI with brief applications in ‘basket’ and ‘flower’ configurations, reducing LADT and supporting efficient same-session PV ablation and appendage occlusion.^1,3^

In redo settings without organized atrial tachycardias, high-resolution 3D mapping for gap identification may be omitted when prioritizing LADT minimization in combined procedures, as supported by the Farapulse system's efficacy in empirical wide-antral re-isolation Pre-procedural reconnection of all PVs was confirmed via diagnostic catheter potentials, enabling standardized PFA delivery. This facilitated safe single-session LAAO via the same transseptal access, reducing cumulative vascular and thrombotic risks.^1,3^

Although PFA generally demands less stringent contact compared to RF, maintaining adequate contact is still crucial for effective results. PFA catheters are also notably easier to manoeuvre within the LA, particularly when employing transseptal puncture techniques optimized for LAAO, which typically requires a lower and more posterior puncture.^2,4^ When a single transseptal puncture is used, priority is generally given to LAAO device delivery, even if this is suboptimal for ablation.^4^ In this case, no significant ridge oedema or changes in LAA ostial dimensions were observed on post-ablation TOE.

The decision to perform PFA prior to LAAO was based on procedural anatomy: PFA-first reduces catheter-device interaction risk, if repeated catheter exchanges are anticipated. Conversely, LAAO-first may be advantageous if significant post-PFA ridge oedema is anticipated, allowing device sizing before swelling develops. Avoiding a second venous access site also reduces bleeding risk.

This case exemplifies an unusual indication for combined PFA and LAAO: severe adenomyosis-related menorrhagia refractory to medical management in a patient with competing thrombotic and bleeding risks. First-line hormonal therapies were either contraindicated or ineffective in the context of recent coronary stenting requiring dual antiplatelet therapy plus anticoagulation. Unlike guidelines addressing menorrhagia in general populations with low thrombotic risk, this multidisciplinary heart-team decision prioritized elimination of systemic anticoagulation exposure, using LAAO as an alternative stroke-prevention strategy uniquely suited to this patient's bleeding phenotype.

PFA has been shown to cause less endothelial damage and tissue oedema compared to RF ablation, potentially reducing the risk of LAA under-sizing, device embolization, and peri-device leaks.^5^

The choice between combined single-session PFA and LAAO vs. staged procedures merits discussion. Combined procedures offer procedural efficiency (single anaesthesia, hospitalization, vascular access), reducing overall procedural burden and cumulative radiation exposure.^4^ Staged approaches permit optimized PVI confirmation before sizing LAAO and allow post-PFA ridge oedema resolution prior to device implantation, potentially improving device stability. However, staged procedures double patient exposure to anaesthesia and vascular intervention. The OPTION trial subgroup analyses found no superiority of concomitant over sequential LAAO, supporting both strategies as valid.^5^ Regarding AF recurrence, studies indicate that combined procedures achieve similar reductions in AF burden over 2 years compared to catheter ablation alone.^9,10^

Emerging data, including the OPTION trial,^5^ demonstrate that PVI with concomitant Watchman FLX implantation is non-inferior in preventing major adverse events and superior in reducing long-term bleeding compared to continued anticoagulation.

Our case exemplifies these benefits: PFA facilitated efficient PV and posterior wall isolation, enabling same-session Watchman FLX implantation without complications. One-year follow-up revealed no AF recurrence, device-related thrombosis, pericardial effusion, or peri-device leak. The unusual gynaecological indication—adenomyosis-related haemorrhage in a patient ineligible for both prolonged anticoagulation and hormonal therapy—highlights an expanding role for combined PFA and LAAO beyond traditional indications.

In conclusion, single-session PFA and LAAO appears to be a viable option for selected AF patients who require stroke prevention but cannot tolerate prolonged anticoagulation. While emerging evidence strongly supports this approach, broader adoption should await further large-scale evaluation.

**Figure 5:**
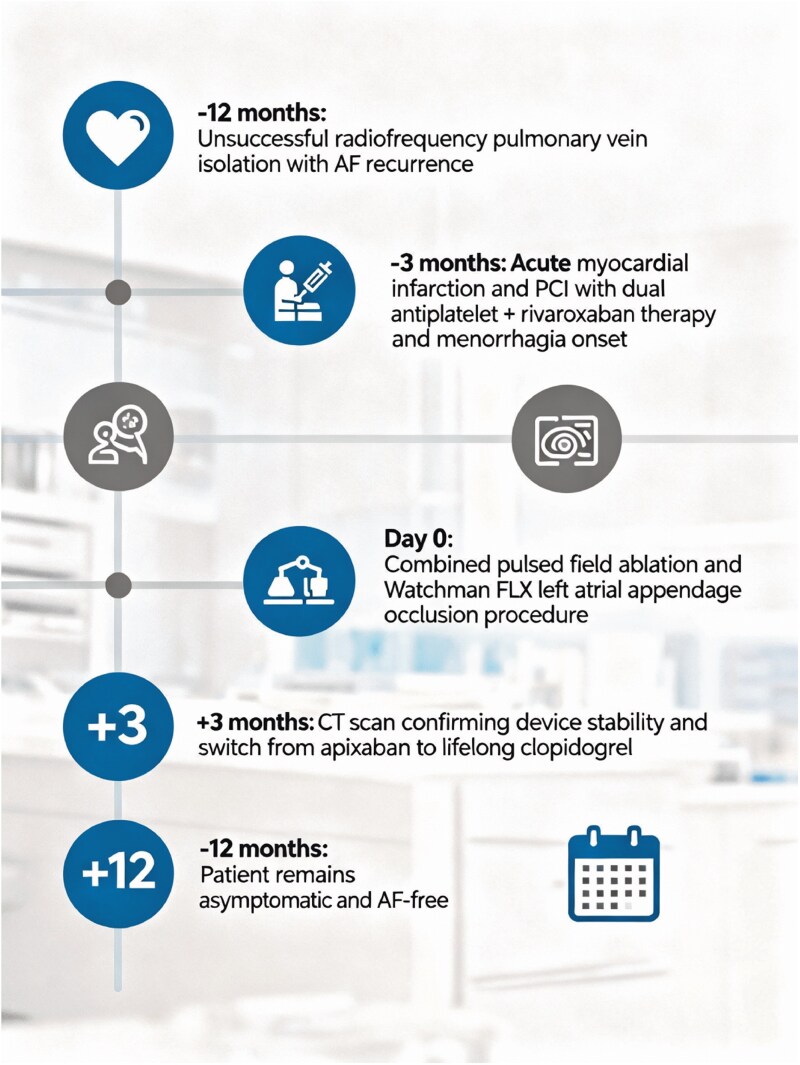
Summary figure -clinical timeline of key events.

The clinical timeline is shown in *Figure 5*.

## Data Availability

The data underlying this article are available in the article and its online Supplementary material. Patient consent forms and procedural videos are available upon reasonable request to the corresponding author.

## Lead author biography

Dr Sophia Alexiou, MD, MSc, is a cardiology resident at Hippokration General Hospital in Thessaloniki, Greece, and recently completed a 6-month electrophysiology fellowship at Ospedale Niguarda in Milan. She holds a Master’s degree in Biomedical Research Methodology and actively participates in international cardiology congresses and ongoing clinical research initiatives.

## Notes

**Consent:** Patient consent was secured using the European Heart Journal—Case Reports consent form, in accordance with COPE guidelines.
